# Design of Functional Powdered Beverages Containing Co-Microcapsules of Sacha Inchi *P. huayllabambana* Oil and Antioxidant Extracts of Camu Camu and Mango Skins

**DOI:** 10.3390/antiox11081420

**Published:** 2022-07-22

**Authors:** Nancy Chasquibol, Rafael Alarcón, Billy Francisco Gonzales, Axel Sotelo, Lourdes Landoni, Gabriela Gallardo, Belén García, M. Carmen Pérez-Camino

**Affiliations:** 1 Grupo de Investigación en Alimentos Funcionales, Carrera de Ingeniería Industrial, Instituto de Investigación Científica, Universidad de Lima, Av. Javier Prado Este 4600, Fundo Monterrico Chico, Surco, 15023 Lima, Peru; ralarcor@ulima.edu.pe (R.A.); bgonzale@ulima.edu.pe (B.F.G.); alex_94sc@hotmail.com (A.S.); 2 Instituto Nacional de Tecnología Industrial (INTI), Av. Gral Paz 5445, San Martín B1650, Argentina; llandoni@inti.gob.ar; 3 Instituto Nacional de Tecnología Agropecuaria (INTA), Gabriel de Aristizabal, William C. Morris, Buenos Aires B1686, Argentina; gallardo.gabrielal@inta.gob.ar; 4 Instituto de la Grasa-Consejo Superior de Investigaciones Científicas, Campus Universidad Pablo de Olavide Ed. 46, Crtra. Sevilla-Utrera km 1, 41013 Sevilla, Spain; belgarpez@ipb.csic.es (B.G.); mcperezcamino@ig.csic.es (M.C.P.-C.)

**Keywords:** antioxidant activity, co-microencapsulation, phenolic compounds, microencapsulation, ultrasound–microwave-assisted extraction, oxidative stability, sacha inchi, *Plukenetia huayllabambana* oil, functional powdered beverage

## Abstract

Sacha inchi *Plukenetia huayllabambana* oil (SIPHO) was co-microencapsulated, by spray drying using gum arabic as a coating material, with antioxidant extracts of camu camu (*Myrciaria dubia* (HBK) McVaugh) (CCSE) and mango (*Mangifera indica*) (MSE) skins obtained by ultrasound–microwave-assisted extraction (UMAE). The physicochemical characteristics of the microcapsules, such as, particle size, morphology, and moisture, as well as the encapsulation efficiency, the fatty acid composition, and oxidative stability, were determined in order to select the best formulation for the design of functional powdered beverages. The formulation with the highest amounts of ω3 acids and polyphenols was used to prepare a functional powdered beverage that contained ω3 (52.74%), antioxidant activity (324.80 mg AAE/100 g powder), and acceptable sensory attributes.

## 1. Introduction

It is well known that a great variety of foods have some special benefits for human health. These kinds of foods have been named functional foods, used for the first time in Japan in the 1980s [1]. Functional foods were defined as processed or natural foods that, in addition to providing basic nutrition, could have other positive or beneficial effects on health [1]. Several plant species have different bioactive compounds that provide their bioactive capacities such as antioxidant, antihypertensive, hypoglycemiant, and others. For example, yellow, orange, and green leafy vegetables and fruits provide great quantities of β-carotene, an important antioxidant molecule for reducing eye diseases [2]. Other kinds of bioactive compounds are polyphenols, which are useful as antioxidants for the prevention and treatment of cardiovascular diseases, obesity, diabetes, etc. [3].

Peruvian biodiversity has been recognized as a great source of endemic plant species with bioactive potential. Fruits, such as cherimoya, lucuma, sauco, or sweet pepino; roots and tubercules such as yacon, potatoes, or yam; and seeds such as quinoa, amaranth, or tarwi, can be highlighted [4]. Camu camu (*Myrciaria dubia* (H.B.K.) MC Vaugh) and mango (*Mangifera indica*) skins have been demonstrated to have high antioxidant activities.

Camu camu is a fruit from a shrub that grows mainly throughout the Amazon rainforest of Peru, Colombia, Venezuela, and Brazil.

Mango is cultivated in many countries such as Pakistan, Bangladesh, Thailand, Malaysia, USA, Venezuela, Mexico, Brazil, and Peru [5]. The main mango varieties grown in Peru are red: Edward, Haden, Kent, Tommy Atkins, Zill; green: Keitt, Amelia, Julie, Alphonse, and yellow: Ataulfo, Manila Super, Nam Doc Mai [6]. They are grown in Piura, Lambayeque, Ancash and Casma [7].

Camu camu is recognized as a source of phenolic compounds. They belong to several subclasses such as flavonols, ellagitannins, ellagic and gallic acid derivatives, anthocyanins, and proanthocyanidins [8]. At present, to determine the identity of the main anthioxidants of camu camu, techniques such as HPLC-PDA, HPLC-MS/MS, or ^1^H NMR are used. The major pigments identified in this fruit are cyanidin-3-glucoside, followed by delphinidin-3-glucoside [9]. The camu camu fruit is rarely eaten fresh, as it is processes to obtain juices. Also, from the fruits are obtained concentrates and capsules, since they are an important source of vitamin C. As a result of their processing, a large volume of waste is generated, which represents around 42% of the weight of the fruit [10].

The mango fruit, due to its unique flavor, fragrance, and texture, is one of the most recognized tropical fruits in the world and enjoys the status of “the king of fruits” [11]. Besides, the mango fruit provides the human diet with macro and micronutrients and with a large number of bioactive compounds. As a result, it has been assigned as a functional food [12]. Even more, other parts of the mango plant, apart from the pulp, skin, and pit of its fruit, such as the leaves and bark, are good sources of bioactive compounds, fiber [13,14], and antioxidants with the ability of neutralize free radicals, which are responsible for many degenerative diseases [15].

With respect to the plant of sacha inchi, *Plukenetia huayllabambana*, also known as ‘Giant Sacha Inchi’ or ‘Giant Inca Peanut’, it is native of the tropical rain forest, in the Amazon region of South America, to be more precise, it grows in the province of Rodriguez de Mendoza, Department of Amazonas, Peru. The *P. huayllabambana* can be considered as a plant of pharmacological value for its potentially bioactive components, including fatty acids, phytosterol, and diterpene alcohol [16]. The seeds have an oil yield between 30.3–41.2%, and the oil contains a high percentage of ω3 fatty acids (55.62 to 60.42% α-linolenic acid (Ln)) and is characterized by its singular campesterol/stigmasterol ratio (1:6) [17]. The ω3 fatty acids have a high susceptibility to oxidation due to their chemical structure [18]. To delay or inhibit its deterioration, it is applied to an alternative emerging technology, microencapsulation [19]. This consists of the preparation of an emulsion of oil-in-water with encapsulating agents such as fibers, gums, proteins, or carbohydrates, and their subsequent drying.

The microencapsulation of sacha inchi oil prevents it from losing more than 6% of the ω3 content and could raise the shelf life of the sacha inchi oil from *P. huayllabambana* and *P. volubilis* to 89.8% and 213.7%, respectively [20,21]. The encapsulating agents that have the better characteristics to provide oxidative stability for microcapsules of sacha inchi oil (*P. huayllabambana* and *P. volubilis*) are Hi-Cap^®^ modified starch, followed by gum arabic, maltodextrin, and whey protein concentrate [22].

With the purpose of palliating certain malnutrition states due to a deficiency of ω3 fatty acids, phytosterols, and antioxidants, we proposed the design of a functional food, easy to prepare and ingest taking advantage of some fruit by-products and raw materials from Peruvian biodiversity. Therefore, aim of this work was to develop a functional powdered beverage with co-microcapsules of sacha inchi (*Plukenetia huayllabambana*) oil and antioxidant extracts of camu camu (*Myrciaria dubia* (H.B.K.) Mc Vaugh) and mango (*Mangifera indica*) skins, and comparing the physicochemical characteristics such as moisture, total and surface phenolic content, antioxidant activity, lipid profile, hygroscopicity, solubility, density, bioavailability, and the oxidative stability of the beverage formulations in order to select the best.

## 2. Materials and Methods

### 2.1. Raw Material

Sacha inchi seeds from the species *Plukenetia huayllabambana* were collected in the province of Rodriguez de Mendoza, Department of Amazonas, Peru. The oils from sacha inchi seeds were obtained by a cold-pressed system in the Functional Foods Laboratory from the Universidad de Lima, Peru and kept at 4 °C sheltered from light until use. The camu camu (*Myrciaria dubia* (H.B.K.) MC Vaugh) (CCS) and mango (*Mangifera indica* var. Edward) (MS) skins were obtained in the local market of Lima city, Peru. The fruits were washed and dried by infrared dryer (IRC D18, Irconfort, Sevilla, Spain) at 40 °C, then ground in a food shredder (Grindomix GM200, Restch, Haan, Germany) and kept at −5 °C in polyethylene bags prior to phenolic extraction. Gum arabic (GA) was acquired from Frutarom, Peru S.A. The extraction of phenolic compounds from camu camu and mango dried skins was performed using an ultrasound–microwave-assisted extraction (UMAE) (CW-2000, Nade, Shanghai, China) [23]. The optimal UMAE conditions were previously determined as follows: solid/solvent ratio was 6/100 (g/mL), the solvent was ethanol (50%) at 2.5 pH. Temperatures and times of extraction were settled at 25 °C for 35 min, and 40 °C for 40 min, the microwave and ultrasound power were 500 W and 50 W, respectively. The resulting extracts were evaporated at 30 °C using a rotary evaporator (Buchi rotavapor R 100, BUCHI Labortechnic AG, Flawil, Switzerland). The extract was reserved at 5 °C until subsequent determinations and processes.

#### Total Phenolic Content (TPC) of Antioxidants Extracts

The total phenolic content (TPC) was determined by the Folin–Ciocalteau method previously reported [23,24]. A total of 0.2 mL of dry skin extract was dissolved in 8 mL of water and 0.5 mL of Folin–Ciocalteau (2 N), and the mixture was stirred using a vortex for 1 min. After 6 min, 1.5 mL of sodium carbonate solution (20%) was added and it was left to rest for 30 min protected from light. The absorbance of the solution was measured at 760 nm using a spectrophotometer (1205 Vis Spectrophotometer UNICO, Dayton, NJ, USA). Ultrapure water was used as a control blank. The results were expressed as µg of gallic acid equivalent (GAE)/mL skin extract. All analyses were carried out in triplicate and the results expressed as mean values.

### 2.2. Co-Microencapsulation of Antioxidant Extracts with Sacha Inchi (P. huayllabambana) Oil

A total of five samples of microcapsules were prepared by mixing phenolic camu camu and/or mango skin extracts (CCSE, MSE) with gum arabic (GA) and sacha inchi *P. huayllabambana* oil (SIPHO) (Table 1). The emulsions were prepared with GA as the coating material and distilled water containing camu camu and mango skins extracts (180 g). Then, the SIPHO was added at a concentration of 18% with respect to total solids [25]. The optimal conditions were previously determined. To obtain homogenous emulsions a Silverson homogenizer L5M-A-England, operating at 9000 rpm for 10 min was used. The feed mixtures were spray-dried in a Büchi B-290-Switzerland with a nozzle atomization system (0.7 mm nozzle diameter). The inlet and outlet air temperatures were 140 °C and 70 °C, respectively, and the optimal feed flow rate was established at 55 mL/min. The flow rate of the drying air was 55 m^3^/h and 0.06 MPa the compressor air pressure. For further analysis, the final dried powders collected were stored in opaque hermetic bags at −5 °C.

#### 2.2.1. Moisture Determination

The moisture of the microcapsules was determined by gravimetry, drying until constant weight using a halogen moisture analyzer (KERN DBS 60-3 halogen balance, KERN & Soohn GmbH, D-72336 Balingen, Germany) at 103 ± 2 °C. A quantity of 1 g of microcapsules was placed on the plate of the halogen balance, and the loss-weight was registered until it reached a constant value that corresponds to the weighed sample moisture. The determinations were performed in triplicate.

#### 2.2.2. Encapsulation Efficiency (EE)

The encapsulation efficiency (EE) was determined by obtaining the non-encapsulated SIPHO following a method previously reported [26]. In a glass jar with a lid, 2.5 g of microcapsules is weighed. Next, thirty-five mL of diethyl ether were added and the mixture was shaken using a magnetic stirrer for 1 min at 25 °C. Then, the mixture was filtered through Whatman filter paper N°1, and the powder was washed with 15 mL of diethyl ether. Finally, the solvent was evaporated under vacuum and nitrogen at 35 °C. The non-encapsulated SIPHO obtained was weighed to calculate the encapsulation efficiency (EE).

The extraction procedure of total oil was based on a method already reported [27,28]. Five g of microcapsules was added to 40 mL of ultrapure hot water (65 °C) and slightly stirred, then 8 mL of NH_4_OH 30% (*w*/*w*) was added and the mixture was kept under stirring for 15 min, maintaining a constant temperature. Next, the samples were cooled at room temperature, and the lipids were extracted by three liquid–liquid extractions using ethanol, diethyl ether, and hexane, following exactly the next sequence: firstly, 20 mL of ethanol, then, 50 mL of diethyl ether, and 50 mL of hexane. After the addition of each solvent, the mixture is stirred. A second extraction is conducted with 25, 25, and 10 mL of diethyl ether, hexane, and ethanol respectively, and a third extraction with 25 mL of each, diethyl ether and hexane. Ten additional mL of ethanol is added when emulsions appeared. The collected extracts were filtered through anhydrous Na_2_SO_4_ using Whatman N°1 filter paper, and subsequently evaporated using a rotary evaporator under a stream of nitrogen to remove the solvent and to obtain a constant weight.

Extraction of the surface oil (SIO) for the powdered beverage was determined with 5 g of samples [28], 50 mL of hexane was added and stirred by means of a magnetic stirrer for 10 min at room temperature. Immediately the extract was filtered on Whatman N°1 filter paper, the solvent was evaporated by means of a rotary evaporator, and it was finally dried by means of a nitrogen stream until constant weight.

The encapsulation efficiency (EE) was determined taking into account the total oil added to form the emulsion (TO), which is placed at the surface SIO and core contained within the microcapsules, following Equation (1):

(1) $$\mathrm{EE}\;\left(\%\right)=\frac{\left(\mathrm{TO}\;-\;\mathrm{SIO}\right)}{\mathrm{TO}}\times 100$$

#### 2.2.3. Fatty Acid Composition

Fatty acid methyl esters (FAMEs) were prepared according to a method previously reported [29]; 50 mg of the oil samples weighed in a 4 mL test tube, 2 mL of hexane, and 0.5 mL of 2 N KOH in methanol were added. The FAMEs formed were placed in a 2 mL chromatographic vial, and 1 µL was injected and analyzed by gas chromatography (GC 7890B Agilent Technologies, Santa Clara, CA, USA). The separation was performed using a SP2380 polar capillary column (poly (90% biscyanopropyl–10% cyanopropylphenyl) siloxane) of 60 m length × 0.25 mm i.d. and 0.20 µm film thickness. The injector and detector (FID) temperatures were 225 and 250 °C, respectively, and hydrogen was used as the carrier gas at a flow rate of 1.0 mL/min. The temperature of the oven was 165 °C, and increased at 3 °C/min up to 230 °C, maintaining this temperature for 2 min.

#### 2.2.4. Tocopherol Analysis

The tocopherol concentrations were determined by HPLC-FL [30]. The procedure was as follows: 50 mg of the extracted oil was weighed in a 10 mL volumetric flask that was filled with hexane of chromatographic quality, to be immediately analyzed by HPLC equipped with a normal phase silica column and a fluorescence detector. The wavelengths were 290 nm and 330 nm for excitation and emission, respectively.

#### 2.2.5. Particle Size Distribution and Morphology

The particle size distribution was determined according to the method previously reported [22] by laser diffraction spectroscopy on MasterSizer Microequipment (Malvern Panalytical, Malvern, United Kingdom). The microcapsules, suspended in deionized water, were sonicated in a bath for 2 min and immediately measured. De Broukere mean diameter (D4,3) or volume-weighted mean size was obtained. Photomicrographs of samples were obtained using a FEI scanning electron microscope (QUANTA 250 FEG, Hillsboro, OR, USA). Previously, samples were gold sputtered with an Edwards Sputter Coater S150B at 5 kV (Kings Grove Industrial Estate, United Kingdom).

#### 2.2.6. Total Phenolic Content (TPC) and Surface Phenolic Content (SPC)

Total phenolic content (TPC) in the microcapsules was determined by the Folin–Ciocalteau method [23,24,31]. A total of 15 mg of microcapsules were dissolved in 4.5 mL of methanol and then stirred using a vortex (VELP Scientifica, Usmate Velate, Italy) for 1 min. Next, 2.5 mL of Folin–Ciocalteau reagent 0.2 N was added by vortexing for 1 min. After 5 min, 2 mL of sodium carbonate solution (20%) was added and the content was mixed again and left at 80 °C for 20 min. The mixture was cooled and filtered through Whatman filter paper N°2. The absorbance of the solution was measured at 760 nm by spectrophotometry (1205 Vis Spectrophotometer, UNICO, Dayton, NJ, USA). Ultrapure water was used as a control blank. The results were expressed as µg of gallic acid equivalent (GAE) per gram of microcapsules (powder). All analyses were performed in triplicate and the results are expressed as mean values.

In the case of the surface phenolic content (SPC) of the microcapsules, 4.5 mL of methanol was added to a quantity of 24 mg and this was stirred with a vortex for 1 min and then filtered through Whatman filter paper N°2. The SPC was measured as described for TPC determination. The efficiency, in percentage, of TPC microencapsulation was determined by Equation (2):

(2) $$\mathrm{Polyphenol}\;\mathrm{Encapsulation}\;\mathrm{Efficiency}\;\left(\mathrm{PEE}\right)\;\left(\%\right)=\frac{\left(\mathrm{TPC}\;-\mathrm{SPC}\right)}{\mathrm{TPC}}\times 100$$

#### 2.2.7. Antioxidant Activity by DPPH Radical

The antioxidant activity for microcapsules was determined using the DPPH method [24,32,33]. Fifteen mg of microcapsules were resuspended in 4.5 mL of methanol/acetic acid/water (50:8:42, *v*/*v*/*v*), then stirred for 1 min and left at 80 °C for 20 min. Next, 3.9 mL of 25 ppm DPPH radical solution in MeOH was added, and the samples left in the dark at 25 °C. The mixture was stirred using a vortex for 1 min and then filtered through Whatman filter paper N°2. The absorption of samples was measured at 517 nm (Abs517_sample_) after 1 h of incubation in the dark. For the control sample (blank), 500 µL methanol was mixed with 3.9 mL of 25 ppm DPPH radical solution and left in the dark at 25 °C. The absorbance was measured (Abs517_control_) at 517 nm by employing a spectrophotometer (1205 Vis Spectrophotometer UNICO, USA). All analyses were carried out in triplicate. The percentage inhibition (% I) of free radicals was calculated using Equation (3):

(3) $$\left(\%\;I\right)=\frac{\left(\mathrm{Abs}517\;\mathrm{control})-(\mathrm{Abs}517\;\mathrm{sample}\right)}{\mathrm{Abs}517\;\mathrm{control}}\times 100$$

#### 2.2.8. Oxidative Stability

The oxidative stability of microcapsules was evaluated by differential scanning calorimetry (DSC) and Rancimat^©^ according to procedures previously reported [18,22]. For DSC, the oxidation onset temperature (OOT), defined as the thermal jump associated with an exothermic reaction due to the initial oil oxidation, was determined using a Mettler 20 (Mettler Toledo, Columbus, OH, USA). The ASTM E2009-08 Method A was followed using oxygen as gas, at 50 mL/min; the initial and final temperatures were 25 and 300 °C, respectively, at a speed of 10 °C/min. The induction period (IP), defined as the time required to produce a marked increase in the conductivity, was determined using an 892 Professional Rancimat^©^ (Metrohm, Herisau, Switzerland). To calculate the shelf life at 25 °C, induction periods at 70, 80, 90, and 100 °C for sacha inchi oil, both bulk and microencapsulated, were determined. The air flow was stated at 20 L/h. The determinations were performed in triplicate. The shelf life for all the treatments at 25 °C was calculated using the software of the equipment, by Equation (4):

(4) $$\mathrm{Shelf}\;\mathrm{life}=A\times e^{\left(B\times T\right)}$$

where *T* is the temperature at which the induction period is calculated, and *A* and *B* are the regression coefficients based on the IP determinations.

### 2.3. Development of Functional Powdered Beverage

Five formulations of functional powder beverage were developed (Table 1). The mango powder was mixed with samples of SIPHO microencapsulated. Mango pulp dried by infrared dryer (IRC D18, Irconfort, Sevilla, Spain) at 40 °C for 12 h was ground in the food shredder (Grindomix GM200, Restch, Haan, Germany) using a planetary mixer. The five formulations were kept at −5 °C in polyethylene bags. The ω3 concentration (around 300 mg per size of serving) was taken into consideration for the formulation.

#### 2.3.1. Hygroscopicity and Solubility of Functional Powdered Beverages

The hygroscopicity was determined by placing 1 g of sample in a desiccator containing saturated NaCl solution (75% HReq) at 25 °C [34]. Sample weight was recorded every 20 min for a total of 140 min. The solubility was determined by dissolving 0.5 g of powder in 20 mL of distilled water in a 45 mL volumetric flask and brought to volume. Then it was vortexed for 5 min and centrifuged at 3000 rpm for 5 min; 20 mL of the supernatant was taken and placed in a container that was heated to 105 °C for 2 h. Solubility (%) was calculated by weight difference.

#### 2.3.2. Bulk and Compacted Density and Hausner Index of Functional Powdered Beverages

Bulk and compacted density and Hausner index were determined according to the method previously reported [34]. Bulk density was determined by weighing 1 g of powder into a 10 mL glass cylinder. The volume occupied by the powder is read on the graduated scale. Compacted density was determined using the specimen used in bulk density, but placed on a vortex and stirred for exactly 1 min. Finally, the volume occupied by the powder is read on the graduated scale. The two types of density are expressed in g/mL. The Hausner indez (IH) was determined with Equation (5):

(5) $$\mathrm{IH}=\frac{\mathrm{Compacted Density}}{\mathrm{Bulk Density}}$$

#### 2.3.3. Antioxidant Bioaccessibility of Functional Powdered Beverages

##### Evaluation of Gastrointestinal Simulation

A static system was used for the evaluation of gastrointestinal simulation [35]. The tests were performed in duplicate for each of the treatments. A total of 2.5 g of juice powder was taken, 50 mL of water was added, and the pH was adjusted to 2 with a 6 M HCl solution, and 10% pepsin (Sigma Aldrich^®^ solution ≥ 400 units/mg protein) was added in 0.1 N HCl. Each system was sealed with Parafilm^®^, protected from light with aluminum foil, and kept at 37 °C in a water bath (Vicking brand 305 thermostatic bath) for 2 h with continuous movement (120 movements per min). After that time, it was cooled and the pH was adjusted to 6.5 with 1 M NaHCO_3_ using a pH meter (Metrohm AG CH-9101 Herisau). A solution of pancreatin (4 × USP specifications) 0.4% and porcine bile extract 2.5% were added. The new solution was again sealed and incubated for 2 h at 37 °C with continuous movement. Finally, the system was cooled in an ice bath, the pH of each extract adjusted with a 0.5 M NaOH solution to 7.2, and then centrifuged at 15,000 rpm for 30 min in order to separate the soluble fraction. The fractions were frozen at −25 °C and subsequently lyophilized. The lyophilized extracts were stored at −80 °C until the antioxidant capacity determination. 

##### Evaluation of Antioxidant Activity by TEAC Assay

The antioxidant activity was carried out on the functional beverage powder initial samples, and on the samples after gastrointestinal simulation. The antioxidant activity was determined following the ascorbic acid equivalent antioxidant capacity test (TEAC) [36]. The radical cation ABTS^+^ was produced by reacting ABTS [2,2-azinobis-(3-ethylbenzothiazoline-6-sulfonic acid)] (7 mM) with potassium persulfate (2.45 mM) in ethanol. The mixture was incubated at room temperature in the dark for 12–16 h before use. The ABTS^+^ solution was diluted with ethanol to get an absorbance of 0.70 (±0.02) at 734 nm and equilibrated at 30 °C. Then, to 10 µL aliquots of the samples (juice powder and lyophilized post SGI) reconstituted in ethanol, and to the ascorbic acid standard (0–2000 mM in ethanol) (Fluka Chemie Gmbh. Buchs, Switzerland), 1.0 mL of diluted ABTS^+^ solution was added and absorbances were read at 734 nm at 30 °C, exactly 1 min after initial mixing and up to 6 min thereafter. Percent inhibition of blank absorbance was calculated for each standard ascorbic acid reference, and sample. The results were expressed as mg ascorbic acid equivalent/100 g powder.

#### 2.3.4. Sensory Analysis on Functional Powdered Beverages

The sensory analysis was made according to the method previously reported [37]. A panel of 100 untrained heterogenized panelists (university students and workers between 18–60 years old) evaluated the sensory attributes of the best functional beverage powder formulation reconstituted, for color, smell, taste, and texture. The test was accomplished based on a 10-point hedonic scale and scaled as 1 = *dislike extremely* and 10 = *like extremely*. The panelists received random samples served in plastic containers, and under normal light. Between each sample test they rinsed their mouths with water.

### 2.4. Statistical Analysis

Results were expressed as mean ± standard deviation, all measurements were conducted in triplicate, except for the measurement of oxidative stability and the evaluation of gastrointestinal simulation, which were done in duplicate. Analysis of variance (ANOVA) was used to analyze the acquired data at a 95% significance level; Tukey’s test was used to identify differences between the samples at a 95% confidence level with Minitab 18.0 (Minitab^®^ statistical software, State College, PA, USA).

## 3. Results and Discussion

### 3.1. Total Polyphenols Content

The mixture of camu camu and mango (cv. Edward) skins had the highest TPC (88.50 mg GAE/g), followed by camu camu skin (72.52 mg GAE/g) and mango skin (48.50 mg GAE/g), respectively. In other reports the polyphenol content of mango (cv. Haden) and camu camu skin were 81.24 mg GAE/g skin [38] and 68.97 mg GAE/g [10], respectively. The difference in analytical methods, extraction procedure, and mango variety may have contributed to the differences in phenolic concentration. For both, camu camu and mango peels the main phenols reported are derivatives of the gallic acid. For camu camu the main one is ellagic acid, a catechol derived from the dimerization of gallic acid [8]. In regrad to the mango skin, the major polyphenols are: flavanols (quercetin, kaempferol, and rhamnetin), gallic acid, syringic acid, mangiferin and ellagic acid [12,14]. 

### 3.2. Moisture Determination and Oil Encapsulation Efficiency (OEE) (%) of Microcapsules and Powdered Beverages

According to Table 2, the formulation SIPHO + GA + MSE (220 ppm) retained less moisture content (3.74 ± 0.08%) than the formulation of SIPHO + GA + CCSE (110 ppm) + MSE (110 ppm) (4.32 ± 0.08%), which was similar to that reported by Landoni et al. [22], whereas the functional beverage powder presented moisture contents between 3.15 ± 0.03% for the formulation of SIPHO + GA + CCSE (110 ppm) + MSE (110 ppm) + MP and 3.28 ± 0.15% for the formulation of SIPHO + GA + MP, which is in the specification range (3–4%) for most powders used in the food industry. These low water contents are usually associated with low water activity, which might prevent lipid oxidation [39].

The moisture content of functional beverage powders ranged from 3.15 up to 3.25%, which was similar to that reported by De Beer et al. [40] and Saikia et al. [41] for the functional beverage powder developed with *Cyclopia subternata* and starfruit (*Averrhoa carambola* L.), respectively. Regarding the oil encapsulation efficiency, the samples showed high values, ranging from 94.17% up to 97.65%. These results were slightly higher than the values reported by Landoni et al. [22] for microcapsules of sacha inchi (*P. huayallabambana*) oil (61.1% and 93.3%).

### 3.3. Fatty Acid Composition of Microcapsules and Powdered Beverages

Table 3 indicates that the microcapsules contained high percentages of polyunsaturated fatty acids (i.e., linolenic acid), ranging between 56.56 ± 0.66 and 57.90 ± 0.55. These values show, with respect to the starting SIPHO (58.12 ± 0.55) a loss of ω3 of around 1%, which is not significant statistically. The *trans* fatty acid isomers, absent in the SIPHO, are present in the microencapsulated SIHO formulations in the range of 0.06–0.07%. These results agreed with those reported by Chasquibol et al. [20] and confirms that the microencapsulation by spray drying does not significantly affect the fatty acid content of the sacha inchi oil [42]. The functional beverage powder had a ω3-linolenic acid content that ranged between 50.88 ± 1.04% and 56.12 ± 1.13%, which could mean that 3 g of the microcapsules contain 300 mg of ω3, this being 33.33% of the adequate intake recommended for children according to the Food and Nutrition Board, Institute of Medicine [43]. The fatty acid profile of the initial sacha inchi oil did not detect *trans* isomers, but during the spray drying process minimum percentages of these components are formed, but within acceptable limits. Their content in both microcapsules and functional beverage powder, presented values between 0.05 ± 0.01% and 0.09 ± 0.00%, respectively. These data were lower than the limit of 2 g per 100 g of fat indicated by the European Union [44]. Regarding the rest of the fatty acids, it can be seen that the presence of PM significantly affects the fatty acids profile. The powdered beverages show, with respect to the microencapsulated SIPHO formulations, higher percentages of palmitic acid (5.63–8.69 vs. 4.30–4.88, respectively) and lower linolenic acid (50.88–56.12 vs. 56.56–57.90, respectively).

### 3.4. Tocopherol Analysis of Microcapsules and Powdered Beverages

Between 10–31% of total tocopherols from microcapsules were lost with respect to the initial oil; however, from the functional beverage powder the loss was between 20–25% (Table 4). For the microencapsulated SIPHO oil formulations, the microcapsules from camu camu and mango extracts showed a high value of tocopherol (2387.2 ± 65.3 mg/kg). In contrast, microcapsules without antioxidant extracts presented a lower content of tocopherol (1824.4 ± 22.6 mg/kg). The 220 ppm of the extracts added, of CCSE and/or MSE, do not provide extra tocopherols to that reported by SIPHO, in contrast to the functional beverage powder, which showed the presence of α-tocopherol. Mango (MP) is the main ingredient of the beverage, and α-tocopherol is reported, in the literature, to be its major tocopherol form [45]. Its presence could be responsible for the smaller total tocopherol loss of the functional beverage powder when compared with microencapsulated formulations.

### 3.5. Total Phenolic Content (TPC) and Surface Phenolic Content (SPC) of Microcapsules

The results of TPC and SPC are shown in Table 5. The TPC of formulations were between 357.73 ± 24.28 and 16,788.86 ± 37.68 µg GAE/g powder. The formulations coated with SIPHO + GA + CCSE (110 ppm) + MSE (110 ppm) (16,788.86 ± 37.68 µg GAE/g powder) was found to have the highest TPC compared with the other formulations. The SPC ranged from 6.13 ± 0.03 to 1385.70 ± 47.81 µg GAE/g powder, the highest value was for the formulations coated with SIPHO + GA + CCSE (110 ppm) + MSE (110 ppm) compared with the others. All the microcapsules with antioxidants showed higher TPC and SPC values than the formulations coated with BHT (220 ppm). The microcapsules coated with GA protect the encapsulated material from oxidation due to the emulsifying ability of GA [46]. The SPC coated with SIPHO + GA (6.13 ± 0.03 µg GAE/g powder) was the lowest due to the strong interaction between the core and coating material [47].

The polyphenolic encapsulation efficiency ranged from 90.25 ± 0.96% to 98.28 ± 0.12%. The highest value obtained may be due to the surface-active biopolymer of the gum arabic [48]. The highest values of encapsulation efficiency and the lowest content of SPC could mean both the antioxidant extracts and the sacha inchi oil were mainly contained in the core of the microcapsules. On the other hand, the high oil encapsulation efficiency and polyphenol encapsulation efficiency values show that the spray drying process was adequate to efficiently co-encapsulate both bioactive compounds.

### 3.6. Antioxidant Activity of Microcapsules

As shown in Table 5, antioxidant activity of the microcapsules ranged from 18,282.26 ± 132.79 to 24,461.36 ± 175.39 µg trolox/g powder; the highest values were 24,461.36 ± 175.39, 24,315.15 ± 83.14, and 24,030.17 ± 38.32 µg trolox/g powder. Due to the significant antioxidant activity of tocopherols, present in important amounts in the sacha inchi oils [49], the antioxidant activity of the microcapsules containing only sacha inchi oil (SIPH + GA) was also evaluated. The result was a lower antioxidant activity than those samples fortified with extracts of camu camu, mango, and their mixtures. These differences between the samples with and without antioxidant extracts were around 6000 µg trolox/g powder, this can be explained by the antioxidant activity of the polyphenol compounds in the extracts. Tolun et al. [50], reported that DPPH antioxidant activity readings show a linear decrease under increased temperature from 160 °C to 180 °C, and that the optimum temperature for encapsulation of grape pomace extract was 140 °C. Other studies suggested that polyphenols are heat labile and prolonged heat treatment may cause irreversible chemical changes to the phenolic content [51]. The antioxidant activity results expressed as percentage of inhibition (%) reported in Table 5 ranged from 75.29 I% to 91.76 I%. There were no significant differences between the microcapsules with fruit antioxidants, but there were differences regarding the microcapsules without antioxidant extracts. 

### 3.7. Particle Size Distribution and Morphology of Microcapsules

According to particle size determination (Table 6), the D4,3 of microcapsules was between 1.6 and 20.9 µm, and were the highest values for microcapsules with camu camu skin extract (CCSE). All samples show a monodisperse distribution. The microcapsules without antioxidant extracts showed similar values to those reported by Landoni et al. [22] for sacha inchi (*P. huayllabambana*) oil microcapsules with gum arabic as wall material (2.6 µm). Alcântara et al. [52], found a particle size between 9.72–9.65 µm for chia oil, which is similar to sacha inchi oil, emulsified with rotor-stator technology at 15,000 rpm and this could explain the higher size presented in this research.

**Figure 1:**
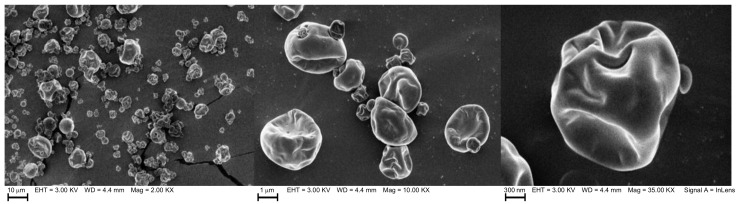
Scanning electron microscopy (SEM) from microencapsulated oil of sacha inchi (*P. huayllabambana*) with GA +CCSE (110 ppm) + MSE (110 ppm).

SEM microscopy, used for the morphological analysis, shows the presence of rounded and concave microcapsules (Figure 1), as expected when the samples are obtained using a spray dryer [53]. Thus, depending on their chemical composition all the microcapsules vary their shape. These results were concordant with Sánchez-Reinoso and Gutiérrez [48], who showed that microencapsulation of sacha inchi oil by spray drying produced irregular microparticles with spherical shapes and some wrinkles. On the other hand, this research found more agglomeration than that reported by Landoni et al. [22]. Furthermore, Alcântara et al. [52] declared that, in general, the spray drying process creates spherical and rounded shape microcapsules, probably due to the homogenization effect of the emulsification stage.

### 3.8. Oxidative Stability of Microcapsules

Regarding the oxidative stability, the results of antioxidant effect of the camu camu and mango skin extracts on the sacha inchi (*P. huayllabambana*) oil microencapsulated using gum arabic as wall material are shown in Table 7. The results obtained were compared with microcapsules of sacha inchi (*P. huayllabambana*) and BHT in the maximum allowed concentration (200 ppm) according to current legislation and were ranged between 173 and 198 °C. The OOT of the microcapsules with antioxidants (188–198 °C) were higher than the microcapsules with BHT (174 °C) and much higher than the microcapsules with only sacha inchi (*P. huayllabambana*) oil (173 °C). These data agree with the results published by Landoni et al. [22], who obtained for sacha inchi (*P. huayllabambana*) oil microcapsules with arabic gum a value of 174 °C. Therefore, the following order can be proposed regarding the protection of the oil under the accelerated oxidation conditions analyzed: SIPHO microcapsules + GA + CCSE > SIPHO microcapsules + GA + CCSE + MSE > SIPHO microcapsules + GA+ MSE > SIPHO microcapsules + GA + BHT > SIPHO microcapsules + GA. Evidently the antioxidants of camu camu and mango skins had a better performance in protecting against oxidation than the synthetic antioxidant BHT.

The lifetime determined by the Rancimat method (Table 8) shows an extrapolated shelf life at 25 °C of between 1616 and 2616 h. According to Alarcón et al. [21], microencapsulation improved the shelf life of sacha inchi oil by 89.8%, however, this research found improvements in shelf life greater than 100%, as in the case of microcapsules with camu camu extract (CCSE, 123% regarding sacha inchi oil shelf life), microcapsules with extract of mango (MSE, 121%, regarding sacha inchi oil shelf life), and microcapsules with extract of camu camu with mango (MSE + CCSE, 103%, regarding sacha inchi oil shelf life). The following order was obtained: SIPHO microcapsules + GA + CCSE > SIPHO microcapsules + GA + MSE > SIPHO microcapsules + GA + CCSE + MSE > SIPHO microcapsules + GA + BHT > SIPHO microcapsules + GA. It can again be concluded that camu camu and mango skins were better antioxidants than the commercial antioxidant BHT.

### 3.9. Physical Properties, Hygroscopicity, and Solubility of Powdered Beverages

All the formulations had a compacted density of 0.67 g/mL and a Hausner index of 1.34. These values indicate that the powdered beverage has little fluidity and would occupy a smaller volume in its packaging. The solubility values obtained are acceptable (greater than 80%), the formulation SIPHO + GA + MP (91.8%) had the highest solubility, but to improve them, the particle size of the powder could be reduced, and binders used to achieve the instant character or increased solubility that this type of product requires. On the other hand, the materials and the type of packaging used must also be considered, since this factor greatly influences the useful life of the product. These data coincide with Sánchez-Reinoso and Gutiérrez [54], who affirmed that microcapsules of sacha inchi (*P. volubilis*) oil present little fluidity due to the higher oil content on the surface of the microcapsule.

### 3.10. Bioavailability Tests of Powdered Beverages

Regarding the antioxidant activity (ABTS), the functional powdered beverage formulations ranged from 287.09 up to 415.54 mg AAE/100 g powder. Other authors also showed functional drinks containing higher antioxidant activity such as Queiroz et al. [55] (9.58–11.26 mg GAE/g powder). The antioxidant bioavailability of the functional beverages showed that samples with dehydrated mango pulp and sacha inchi (*P. huayllabambana*) oil microcapsules + CCSE (110 ppm) + MSE (110 ppm) + GA had almost three times more antioxidant capacity than other formulations, releasing more of its active compounds (65%) compared with the other three formulations (18, 20, and 24%) under similar gastric simulation conditions (Table 9). This fact may be due to a synergistic effect between the antioxidant peel extracts of both fruits [56].

### 3.11. Sensory Analysis of the Best Powdered Beverages

In the sensory evaluation, the acceptability of the powdered beverage formulation SIPHO + GA + CCSE (110 ppm) + MSE (110 ppm) + MP was evaluated. A total of 97% of the panelists recognized the taste of the drink, an acceptability of 88% was obtained and the characteristics of taste, color, smell, and texture had an average value of 7, 9, 7, and 8, respectively, on a scale of 1 to 10. These results show that the functional beverage was sensorily accepted by the panelists. According to Włodarska et al. [57], the sensory aspect would be one of the most important factors influencing eating behavior along with cost, safety, and accessibility.

## 4. Conclusions

Microcapsules of sacha inchi (*Plukenetia huayllabambana*) oil with mango and camu camu skin extracts were successfully prepared using the spray drying method. The microcapsules exhibited a low percentage of moisture content, between 3.74 and 4.32%, high phenolic concentration, 357.73–16,788.86 µg GAE/g power, and also polyphenolic encapsulation efficiency in the range of 90.25–98.28%, and a high antioxidant activity, ranging from 75.29 to 91.76%. They exhibited a composition greatly similar to SIPHO, with a slight loss of ω3 (57 vs. 58%), the presence of *trans* fatty acid isomers (0.06–0.07%), and oil encapsulation efficiency ranged from 94.17 to 95.00%. Therefore, the co-encapsulation process was adequate to microencapsulate both polyphenols and essential fatty acids. Moreover, the antioxidant extracts of natural origin analyzed provide a higher protection to SIPHO against oxidation compared with a synthetic origin antioxidant (BHT) allowed for human consumption, using similar concentrations. Finally, the functional beverage powder (SIPHO + GA + CCSE (110 ppm) + MSE (110 ppm) + MP) designed shows a high content of ω3 (52.74%), 324.80 mg AAE/100 g powder of antioxidant activity, acceptable physicochemical characteristics such as solubility (greater than 80%), an important antioxidant bioavailability of 65%, and great sensory attributes such as color, smell, texture, and taste, measured using a hedonic scale of 1 to 10 points.

## Figures and Tables

**Table 1:** Formulations of sacha inchi *Plukenetia* *huayllabambana* oil (SIPHO) microencapsulated and powdered beverage.

Microencapsulated SIPHO Formulations	Powdered Beverage Formulations
SIPHO + GA	SIPHO + GA + MP ^d^
SIPHO + GA + CCSE ^a^ (220 ppm)	SIPHO + GA + CCSE (220 ppm) + MP
SIPHO + GA + MSE ^b^ (220 ppm)	SIPHO + GA + MSE (220 ppm) + MP
SIPHO + GA + CCSE (110 ppm) + MSE (110 ppm)	SIPHO + GA + CCSE (110 ppm) + MSE (110 ppm) + MP ^d^
SIPHO + GA + BHT ^c^ (200 ppm)	SIPHO + GA + BHT (200 ppm) + MP

^a^ Camu camu skin extract, ^b^ Mango skin extract, ^c^ Commercial antioxidant, ^d^ Mango pulp.

**Table 2:** Moisture (%), surface oil content (SOC) (%) and oil encapsulation efficiency (OEE) (%) of sacha inchi (*P. huayllabambana*) oil (SIPHO) microencapsulated and powdered beverage formulations.

Formulations	Moisture (%)	SOC (%)	OEE (%)
Microencapsulated SIPHO formulations			
SIPHO + GA	3.79 ± 0.28 ^c^	5.83 ± 0.24 ^a^	94.17 ± 0.24 ^a^
SIPHO+ GA + CCSE (220 ppm)	3.88 ± 0.08 ^bc^	5.65 ± 0.92 ^a^	94.35 ± 0.92 ^a^
SIPHO + GA + MSE (220 ppm)	3.74 ± 0.08 ^c^	5.35 ± 0.49 ^a^	94.65 ± 0.49 ^a^
SIPHO+ GA + CCSE (110 ppm) + MSE (110 ppm)	4.32 ± 0.08 ^a^	5.00 ± 0.00 ^a^	95.00 ± 0.00 ^a^
SIPHO+ GA + BHT (200) ppm	4.24 ± 0.08 ^ab^	5.65 ± 0.92 ^a^	94.35 ± 0.78 ^a^
Powdered beverage formulations			
SIPHO + GA + MP	3.28 ± 0.15 ^a^	3.73 ± 0.81 ^a^	96.27 ± 0.81 ^a^
SIPHO+ GA + CCSE (220 ppm) + MP	3.18 ± 0.01 ^a^	3.56 ± 0.91 ^a^	96.44 ± 0.91 ^a^
SIPHO + GA + MSE (220 ppm) + MP	3.19 ± 0.02 ^a^	2.06 ± 0.08 ^a^	97.94 ± 0.08 ^a^
SIPHO+ GA + CCSE (110 ppm) +MSE (110 ppm) + MP	3.15 ± 0.03 ^a^	2.45 ± 0.78 ^a^	97.55 ± 0.78 ^a^
SIPHO + GA + BHT (200 ppm) + MP	3.25 ± 0.06 ^a^	2.35 ± 0.78 ^a^	97.65 ± 0.78 ^a^

Results are expressed as means ± SD (*n* = 3). Means with different superscript lowercase letters (a–c), in the same column, are significantly different (*p* < 0.05).

**Table 3:** Composition in fatty acids (%) of total oil extracted from sacha inchi (*P. huayllabambana*) (SIPHO) microencapsulated and powdered beverage formulations.

Formulations	C_16:0_	C_16:1_	C_17:0_	C_17:1_	C_18:0_	C_18:1_	C_18:2_	C_20:0_	C_18:3_	C_20:1_	*Trans*
Sacha inchi (*Plukenetia huayllabambana*) oil (SIPHO)											
SIPHO	4.50 ± 0.24 ^d^	0.07 ± 0.01 ^d^	0.06 ± 0.01 ^bc^	0.04 ± 0.01 ^b^	1.75 ± 0.05 ^ab^	7.95 ± 0.30 ^d^	26.10 ± 0.50 ^a^	0.31 ± 0.15 ^a^	58.12 ± 0.55 ^a^	0.29 ± 0.15 ^a^	nd *
Microencapsulated SIPHO formulations											
SIPHO + GA	4.55 ± 0.02 ^d^	0.05 ± 0.00 ^d^	0.07 ± 0.00 ^bc^	0.04 ± 0.00 ^b^	1.75 ± 0.02 ^ab^	8.94 ± 0.05 ^cd^	26.70 ± 0.03 ^a^	0.22 ± 0.00 ^a^	57.30 ± 0.12 ^a^	0.23 ± 0.01 ^a^	0.07 ± 0.01 ^a^
SIPHO + GA + CCSE (220 ppm)	4.88 ± 0.14 ^cd^	0.08 ± 0.01 ^d^	0.07 ± 0.00 ^bc^	0.04 ± 0.00 ^b^	1.83 ± 0.01 ^a^	9.31 ± 0.77 ^bcd^	26.60 ± 0.26 a	0.22 ± 0.00 ^a^	56.56 ± 0.66 ^a^	0.25 ± 0.02 ^a^	0.07 ± 0.01 ^a^
SIPHO + GA + MSE (220 ppm)	4.18 ± 0.18 ^d^	0.04 ± 0.01 ^d^	0.05 ± 0.00 ^c^	0.03 ± 0.00 ^b^	1.54 ± 0.09 ^b^	8.50 ± 0.30 ^d^	27.20 ± 0.53 ^a^	0.20 ± 0.15 ^a^	57.90 ± 0.55 ^a^	0.21 ± 0.14 ^a^	0.06 ± 0.01 ^a^
SIPHO + GA + CCSE (110 ppm) + MSE (110 ppm)	4.30 ± 0.18 ^d^	0.05 ± 0.01 ^d^	0.06 ± 0.00 ^bc^	0.03 ± 0.00 ^b^	1.67 ± 0.09 ^ab^	8.72 ± 0.30 ^cd^	27.10 ± 0.53 ^a^	0.21 ± 0.15 ^a^	57.56 ± 0.55 ^a^	0.23 ± 0.14 ^a^	0.06 ± 0.01 ^a^
SIPHO + GA + BHT (200 ppm)	4.84 ± 0.21 ^cd^	0.06 ± 0.00 ^d^	0.06 ± 0.00 ^bc^	0.04 ± 0.00 ^b^	1.80 ± 0.01 ^a^	8.73 ± 0.02 ^cd^	26.90 ± 0.04 ^a^	0.22 ± 0.00 ^a^	56.96 ± 0.15 ^a^	0.27 ± 0.00 ^a^	0.07 ± 0.00 ^a^
Powdered beverage formulations											
SIPHO + GA + MP	7.27 ± 0.04 ^b^	2.09 ± 0.02 ^b^	0.09 ± 0.01 ^bc^	0.10 ± 0.02 ^ab^	1.70 ± 0.01 ^ab^	12.07 ± 0.75 ^a^	24.20 ± 0.22 ^b^	0.20 ± 0.00 ^a^	52.06 ± 0.06 ^b^	0.21 ± 0.00 ^a^	0.09 ± 0.00 ^a^
SIPHO + GA + CCSE (220 ppm) + MP	8.69 ± 0.15 ^a^	3.10 ± 0.22 ^a^	0.08 ± 0.02 ^bc^	0.14 ± 0.02 ^a^	1.73 ± 0.05 ^ab^	10.93 ± 1.79 ^ab^	24.00 ± 0.38 ^b^	0.20 ± 0.00 ^a^	50.88 ± 1.04 ^b^	0.20 ± 0.00 ^a^	0.08 ± 0.00 ^a^
SIPHO + GA + MSE (220 ppm) + MP	7.44 ±0.43 ^b^	2.33 ± 0.24 ^b^	0.06 ± 0.01 ^bc^	0.16 ± 0.07 ^a^	1.61 ± 0.07 ^ab^	10.35 ± 0.07 ^abc^	24.52 ± 0.26 ^b^	0.23 ± 0.04 ^a^	52.88 ± 0.88 ^b^	0.27 ± 0.07 ^a^	0.08 ± 0.00 ^a^
SIPHO + GA + CCSE (110 ppm) + MSE (110 ppm) + MP	7.64 ± 0.55 ^ab^	2.18 ± 0.26 ^b^	0.13 ± 0.00 ^a^	0.17 ± 0.01 ^a^	1.66 ± 0.05 ^ab^	10.42 ± 0.74 ^abc^	24.54 ± 0.12 ^b^	0.22 ± 0.04 ^a^	52.74 ± 0.03 ^b^	0.26 ± 0.05 ^a^	0.05 ± 0.01 ^a^
SIPHO + GA + BHT (200 ppm) + MP	5.63 ± 0.41 ^c^	0.94 ± 0.10 ^c^	0.06 ± 0.00 ^bc^	0.08 ± 0.01 ^ab^	1.61 ± 0.11 ^ab^	9.12 ± 0.28 ^cd^	26.02 ± 0.16 ^a^	0.20 ± 0.01 ^a^	56.12 ± 1.13 ^a^	0.22 ± 0.04 ^a^	0.06 ± 0.0 ^a^

Results are expressed as means ± SD (*n* = 3); * nd = not detected. Means with different superscript lowercase letters (a–d), in the same column, are significantly different (*p* < 0.05).

**Table 4:** Composition in tocopherols (mg/kg) of sacha inchi (*P. huayllabambana*) oil (SIPHO) microencapsulated and powdered beverage formulations.

Formulations	α-Tocopherol	γ-Tocopherol	δ-Tocopherol	Total
Sacha inchi (*Plukenetia huayllabambana*) oil (mg/kg)				
SIPHO	-	1811.0 ± 20.0 ^a^	849.0 ± 15.0 ^a^	2660.0 ± 45.0 ^a^
Microencapsulated SIPHO formulations (mg/kg)				
SIPHO + GA	-	1078.6 ± 8.2 ^g^	745.8 ± 8.2 ^b^	1824.4 ± 22.6 ^e^
SIPHO + GA + CCSE (220 ppm)	-	1355.7 ± 14.0 ^cde^	698.5 ± 10.2 ^bc^	2054.2 ± 24.2 ^d^
SIPHO + GA + MSE (220 ppm)	-	1392.7 ± 10.0 ^cd^	683.4 ± 10.9 ^bcd^	2076.0 ± 20.9 ^ed^
SIPHO + GA + CCSE (110 ppm) + MSE (110 ppm)	-	1636.5 ± 48.1 ^b^	750.7 ± 17.1 ^b^	2387.2 ± 65.3 ^b^
SIPHO + GA + BHT (200 ppm)	-	1475.3 ± 35.9 ^bc^	745.7 ± 25.9 ^b^	2221.0 ± 61.8 ^c^
Powdered beverage formulations (mg/kg)				
SIPHO + GA +MP	243.1 ± 7.6 ^bc^	1129.8 ± 30.9 ^fg^	644.7 ± 32.9 ^bcde^	2017.6 ± 31.4 ^d^
SIPHO + GA + CCSE (220 ppm) + MP	324.6 ± 38.8 ^a^	1208.1 ± 34.0 ^efg^	585.4 ± 27.4 ^e^	2118.1 ± 22.6 ^cd^
SIPHO + GA + MSE (220 ppm) + MP	228.5 ± 7.8 ^bc^	1245.3 ± 13.6 ^defg^	587.5 ±31.8 ^de^	2061.2 ± 53.2 ^d^
SIPHO + GA + CCSE (110 ppm) + MSE (110 ppm) + MP	266.1 ± 2.7 ^b^	1290.2 ± 34.2 ^def^	596.3 ± 15.7 ^de^	2152.5 ± 21.2 ^cd^
SIPHO + GA + BHT (200 ppm) + MP	202.1 ± 17.8 ^c^	1299.2 ± 51.1 ^def^	667.2 ± 45.9 ^bcde^	2136.1 ± 14.8 ^cd^

Results are expressed as means ± SD (*n* = 3). Means with different superscript lowercase letters (a–g), in the same column, are significantly different (*p* < 0.05).

**Table 5:** Total phenolic content (TPC) (μg GAE/g powder), surface phenolic content (SPC) (μg GAE/g powder), polyphenol encapsulation efficiency (PEE) (%), DPPH (µg trolox/g powder) and inhibition (%) of sacha inchi (*P. huayllabambana*) oil (SIPHO) microencapsulated.

Microencapsulated SIPHO Formulations	TPC (μg GAE/g Powder)	SPC (μg GAE/g Powder)	PEE (%)	DPPH (µg trolox/g Powder)	Inhibition (%)
SIPHO + GA	357.73 ± 24.28 ^d^	6.13 ± 0.03 ^d^	98.28 ± 0.12 ^a^	18,513.53 ± 24.42 ^c^	75.29 ± 0.23 ^b^
SIPHO+ GA + CCSE (220 ppm)	11,156.60 ± 82.44 ^b^	613.52 ± 18.12 ^c^	94.50 ± 0.20 ^b^	24,030.17 ± 38.32 ^b^	91.70 ± 0.14 ^a^
SIPHO + GA + MSE (220 ppm)	7422.76 ± 224.82 ^c^	722.19 ± 48.64 ^b^	90.25 ± 0.96 ^c^	24,461.36 ± 175.39 ^a^	91.76 ± 0.09 ^a^
SIPHO + GA + CCSE (110 ppm) + MSE (110 ppm)	16,788.86 ± 37.68 ^a^	1385.70 ± 47.81 ^a^	91.75 ± 0.30 ^bc^	24,315.15 ± 83.14 ^ab^	91.60 ± 0.09 ^a^
SIPHO + GA + BHT (220 ppm)	381.41 ± 23.14 ^d^	34.33 ± 8.83 ^d^	91.01 ± 2.2 ^c^	18,282.26 ± 132.79 ^c^	75.49 ± 0.69 ^b^

Results are expressed as means ± SD (*n* = 3). Means with different superscript lowercase letters (a–d), in the same column, are significantly different (*p* < 0.05).

**Table 6:** Particle size (µm) of sacha inchi (*P. huayllabambana*) oil (SIPHO) microencapsulated.

Microencapsulated SIPHO Formulations	*D* [4,3] µm	Span	Volume Distribution, µm		
			*D* (*v*, 0.1)	*D* (*v*, 0.5)	*D* (*v*, 0.9)
SIPHO + GA	2.6 (0.1)	2.0 (6.1)	0.8 (0.1)	2.1 (0.1)	5.1 (0.1)
SIPHO + GA + CCSE (220 ppm)	20.9 (1.4)	1.1 (0.1)	1.1 (0.1)	6.2 (0.1)	66.4 (0.2)
SIPHO + GA +MSE (220 ppm)	1.6 (0.1)	1.4 (0.1)	0.7 (0.1)	1.4 (0.1)	2.7 (0.2)
SIPHO + GA +CCSE (110 ppm) + MSE (110 ppm)	4.0 (1.4)	1.5 (0.1)	0.7 (0.1)	1.4 (0.1)	2.9 (0.2)

The results correspond to the average and standard deviation indicated in parentheses. *D* [4,3] µm: volume weighted mean size. *D* (*v*, 0.1): particle size for which 10% of the sample is less than that limit. *D* (*v*, 0.5): particle size for which 50% of the sample is less than that limit. *D* (*v*, 0.9): particle size for which 90% of the sample is less than that limit. Span: dispersion index. (Data available on: Alarcon et al. [25]).

**Table 7:** Oxidation onset temperature (OOT) (°C) of sacha inchi (*P. huayllabambana*) oil (SIPHO) microencapsulated.

Microencapsulated SIPHO Formulations	Tangent Method (Onset)		
	OOT (°C)		
	Prom	Ds	U
SIPHO + GA	173	<1	±10
SIPHO + GA + CCSE (220 ppm)	198	<1	±12
SIPHO + GA +MSE (220 ppm)	188	<1	±11
SIPHO + GA +CCSE (110 ppm) + MSE (110 ppm)	192	<1	±11
SIPHO + GA + BHT (200 ppm)	174	4	±11
SIPHO	163	<1	±9

Prom: average value obtained from the OOT of two independent determinations. Ds: Standard deviation. U: expanded uncertainty of the OOT test. “The reported expanded measurement uncertainty was calculated by multiplying the combined standard uncertainty by a coverage factor *k* = 2, which corresponds to an approximate 95% confidence level under normal distribution”.

**Table 8:** Lifetime (h), by Rancimat method, of sacha inchi (*P. huayllabambana*) oil (SIPHO) microencapsulated.

Microencapsulated SIPHO Formulations	Induction Time (h)				Extrapolated Lifetime at 25 °C (h)
	70 °C	80 °C	90 °C	100 °C	
SIPHO + GA	59.56 ± 1.26 ^c^	25.94 ± 1.03 ^d^	12.53 ± 0.37 ^e^	6.39 ± 0.14 ^d^	1616 ± 75 ^b^
SIPHO + GA + BHT (200 ppm)	63.52 ± 1.71 ^c^	30.80 ± 1.02 ^c^	14.67 ± 0.54 ^d^	7.15 ± 0.25 ^d^	1694 ± 44 ^b^
SIPHO + GA +MSE (220 ppm)	104.37 ± 1.08 ^b^	48.81 ± 0.60 ^b^	23.50 ± 0.35 ^c^	12.09 ± 0.39 ^c^	2605 ± 65 ^a^
SIPHO + GA + CCSE (220 ppm)	142.14 ± 13.73 ^a^	51.69 ± 1.31 ^b^	30.28 ± 0.18 ^a^	16.82 ± 0.26 ^b^	2616 ± 275 ^a^
SIPHO + GA + CCSE (110 ppm) + MSE (110 ppm)	139.42 ± 4.42 ^a^	59.32 ± 1.84 ^a^	26.88 ± 1.00 ^b^	20.26 ± 0.85 ^a^	2397 ± 293 ^a^
SIPHO	35.90 ± 2.34 ^d^	11.84 ± 0.48 ^e^	5.64 ± 0.15 ^f^	2.90 ± 0.06 ^e^	1176 ± 136 ^c^

Results are expressed as means ± SD (*n* = 3). Means with different superscript lowercase letters (a–f), in the same column, are significantly different (*p* < 0.05).

**Table 9:** Antioxidant activity (mg AAE/100 g powder) and bioaccessibility (%) of powdered beverage formulations.

Powdered Beverage Formulations	mg AAE/100 g Powder	Mg AA/100 G Digested Residue	Percentage of Digested Antioxidants
SIPHO + GA +MP	369.36 ± 6.46 ^c^	65.61 ± 12.04 ^a^	18%
SIPHO + GA + CCSE (220 ppm) + MP	415.54 ± 31.59 ^d^	82.27 ± 18.46 ^a^	20%
SIPHO + GA +MSE (220 ppm) + MP	287.09 ± 8.86 ^b^	68.05 ± 3.01 ^a^	24%
SIPHO + GA +CCSE (110 ppm) + MSE (110 ppm) + MP	324.80 ± 2.83 ^c^	211.06 ± 16.42 ^b^	65%

Results are expressed as means ± SD (*n* = 3). Means with different superscript lowercase letters (a–d), are significantly different (*p* < 0.05).

## Data Availability

Data is contained within the manuscript.
